# Natural language processing applied to mental illness detection: a narrative review

**DOI:** 10.1038/s41746-022-00589-7

**Published:** 2022-04-08

**Authors:** Tianlin Zhang, Annika M. Schoene, Shaoxiong Ji, Sophia Ananiadou

**Affiliations:** 1 Department of Computer Science, The University of Manchester, National Centre for Text Mining, Manchester, UK; 2 Department of Computer Science, Aalto University, Helsinki, Finland; 3 The Alan Turing Institute, London, UK

**Keywords:** Psychiatric disorders, Disease prevention

## Abstract

Mental illness is highly prevalent nowadays, constituting a major cause of distress in people’s life with impact on society’s health and well-being. Mental illness is a complex multi-factorial disease associated with individual risk factors and a variety of socioeconomic, clinical associations. In order to capture these complex associations expressed in a wide variety of textual data, including social media posts, interviews, and clinical notes, natural language processing (NLP) methods demonstrate promising improvements to empower proactive mental healthcare and assist early diagnosis. We provide a narrative review of mental illness detection using NLP in the past decade, to understand methods, trends, challenges and future directions. A total of 399 studies from 10,467 records were included. The review reveals that there is an upward trend in mental illness detection NLP research. Deep learning methods receive more attention and perform better than traditional machine learning methods. We also provide some recommendations for future studies, including the development of novel detection methods, deep learning paradigms and interpretable models.

## Introduction

Mental illnesses, also called mental health disorders, are highly prevalent worldwide, and have been one of the most serious public health concerns^1^. There are many different mental illnesses, including depression, suicidal ideation, bipolar disorder, autism spectrum disorder (ASD), anxiety disorder, schizophrenia, etc., any of which can have a negative influence on an individual’s physical health and well-being with the problem exacerbated due to Covid-19^2^. According to the latest statistics, millions of people worldwide suffer from one or more mental disorders^1^. If mental illness is detected at an early stage, it can be beneficial to overall disease progression and treatment.

There are different text types, in which people express their mood, such as social media messages on social media platforms, transcripts of interviews and clinical notes including the description of patients’ mental states. In recent years, natural language processing (NLP), a branch of artificial intelligence (AI) technologies, has played an essential role in supporting the analysis and management of large scale textual data and facilitating various tasks such as information extraction, sentiment analysis^3^, emotion detection, and mental health surveillance^4–6^. Detecting mental illness from text can be cast as a text classification or sentiment analysis task, where we can leverage NLP techniques to automatically identify early indicators of mental illness to support early detection, prevention and treatment.

Existing reviews introduce mainly the computational methods for mental health illness detection, they mostly focus on specific mental illnesses (suicide^7–9^, depression^10–12^), or specific data sources (social media^13–15^, non-clinical texts^16^). To the best of our knowledge, there is no review of NLP techniques applied to mental illness detection from textual sources recently. We present a broader scope of mental illness detection using NLP that covers a decade of research, different types of mental illness and a variety of data sources. Our review aims to provide a comprehensive overview of the latest trends and recent NLP methodologies used for text-based mental illness detection, and also points at the future challenges and directions. Our review seeks to answer the following questions:

- What are the main NLP trends and approaches for mental illness detection?
- Which features have been used for mental health detection in traditional machine learning-based models?
- Which neural architectures have been commonly used to detect mental illness?
- What are the main challenges and future directions in NLP for mental illness?

## Search methodology

### Search strategy

A comprehensive search was conducted in multiple scientific databases for articles written in English and published between January 2012 and December 2021. The databases include PubMed, Scopus, Web of Science, DBLP computer science bibliography, IEEE Xplore, and ACM Digital Library.

The search query we used was based on four sets of keywords shown in Table 1. For mental illness, 15 terms were identified, related to general terms for mental health and disorders (e.g., mental disorder and mental health), and common specific mental illnesses (e.g., depression, suicide, anxiety). For data source, we searched for general terms about text types (e.g., social media, text, and notes) as well as for names of popular social media platforms, including Twitter and Reddit. The methods and detection sets refer to NLP methods used for mental illness identification.

**Table 1:** Keywords for literature search.

Category	Keywords
Mental illness (1)	Mental disorder, mental health, mental illness
	Depression, suicide, psychology, insomnia, stress, anxiety, schizophrenia, phobias, PTSD (post-traumatic stress disorder), ASD (autism spectrum disorder), anorexia, bipolar
Data sources (2)	Social media, text, language, posts, notes, interviews, records, survey
	Twitter, reddit, weibo, microblog, facebook, instagram
Methods (3)	Natural language processing, deep learning, machine learning, text mining, text analysis
	Neural network, CNN, LSTM, SVM, tree
Detection (4)	Detect, identify, recognize, predict, prevent, screen, assess, understand
Search query	(1) AND (2) AND (3) AND (4)

The keywords of each sets were combined using Boolean operator “OR", and the four sets were combined using Boolean operator “AND". We conducted the searches in December 2021.

### Filtering strategy

A total of 10,467 bibliographic records were retrieved from six databases, of which 7536 records were retained after removing duplication. Then, we used RobotAnalyst^17^, a tool that minimizes the human workload involved in the screening phase of reviews, by prioritizing the most relevant articles for mental illness based on relevancy feedback and active learning^18,19^.

Each of the 7536 records was screened based on title and abstract. Records were removed if the following exclusion criteria were met: (1) the full text was not available in English; (2) the abstract was not relevant to mental illness detection; (3) the method did not use textual experimental data, but speech or image data.

After the screening process, 611 records were retained for further review. An additional manual full-text review was conducted to retain only articles focusing on the description of NLP methods only. The final inclusion criteria were established as follow:

- Articles must study textual data such as contents from social media, electronic health records or transcription of interviews.
- They must focus on NLP methods for mental illness detection, including machine learning-based methods (in this paper, the machine learning methods refer to traditional feature engineering-based machine learning) and deep learning-based methods. We exclude review and data analysis papers.
- They must provide a methodology contribution by (1) proposing a new feature extraction method, a neural architecture, or a novel NLP pipeline; or (2) applying the learning methods to a specific mental health detection domain or task.

**Fig. 1:**
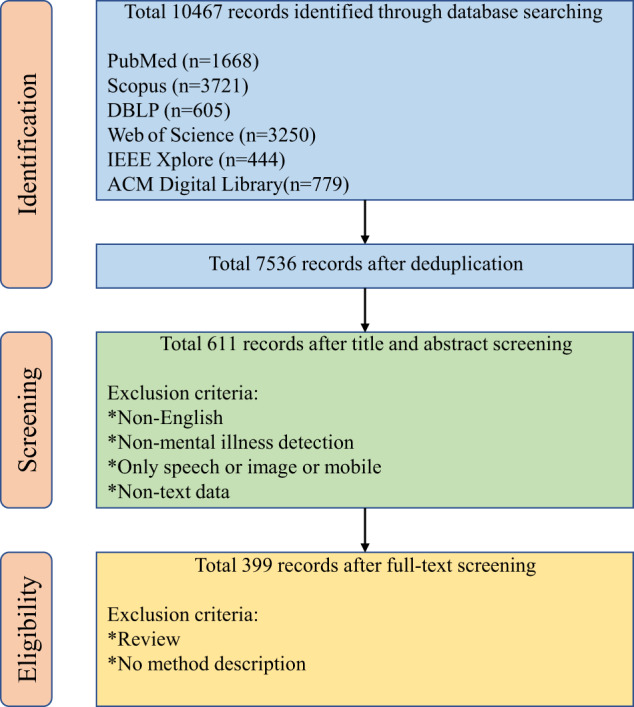
Overview of article selection process. Six databases (PubMed, Scopus, Web of Science, DBLP computer science bibliography, IEEE Xplore, and ACM Digital Library) were searched. The flowchart lists reasons for excluding the study from the data extraction and quality assessment.

Following the full-text screening process, 399 articles were selected. The flow diagram of the article selection process is shown in Fig. 1.

### Data extraction

For each selected article, we extracted the following types of metadata and other information:

- Year of publication.
- The aim of research.
- The dataset used, including type of mental illness (e.g., depression, suicide, and eating disorder), language, and data sources (e.g., Twitter, electronic health records (EHRs) and interviews).
- The NLP method (e.g., machine learning and deep learning) and types of features used (e.g., semantic, syntactic, and topic).

## Findings

**Fig. 2:**
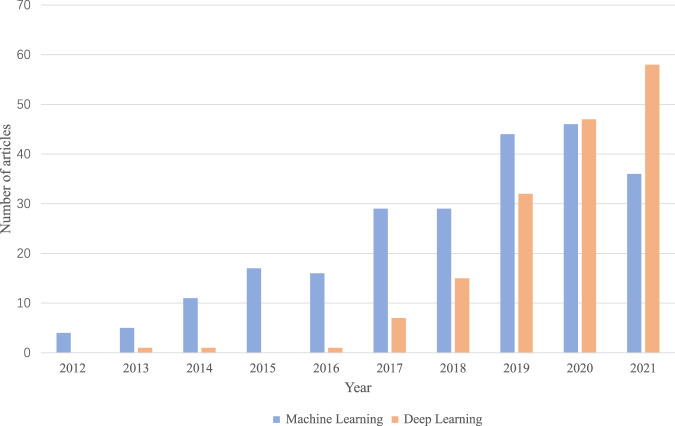
NLP trends applied to mental illness detection research using machine learning and deep learning. The trend of the number of articles containing machine learning-based and deep learning-based methods for detecting mental illness from 2012 to 2021.

We show in Fig. 2 the number of publications retrieved and the methods used in our review, reflecting the trends of the past 10 years. We can observe that: (1) there is an upward trend in NLP-driven mental illness detection research, suggesting the great research value and prospects for automatic mental illness detection from text (2) deep learning-based methods have increased in popularity in the last couple of years.

In the following subsections, we provide an overview of the datasets and the methods used. In section Datesets, we introduce the different types of datasets, which include different mental illness applications, languages and sources. Section NLP methods used to extract data provides an overview of the approaches and summarizes the features for NLP development.

### Datasets

**Fig. 3:**
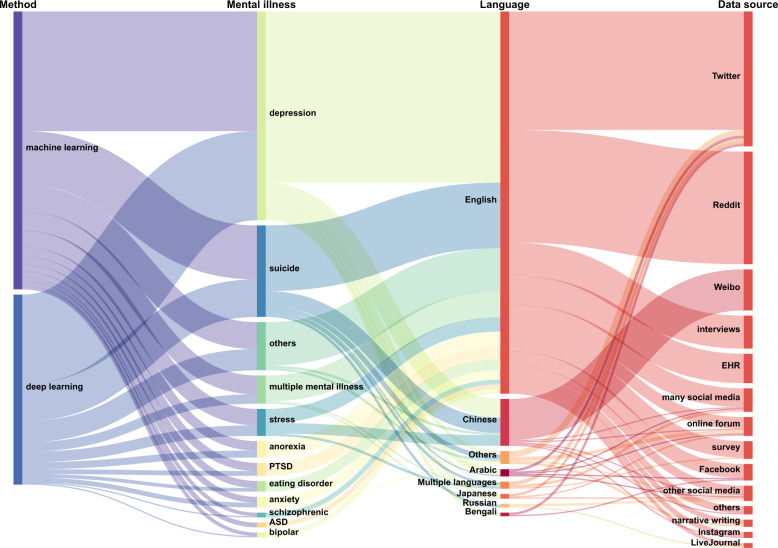
Sankey diagram of NLP methods, illness, languages and applications. The different methods with their associated application are represented via flows. Nodes are represented as rectangles, and the height represents their value. The width of each curved line is proportional to their values.

In order to better train mental illness detection models, reliable and accurate datasets are necessary. There are several sources from which we can collect text data related to mental health, including social media posts, screening surveys, narrative writing, interviews and EHRs. At the same time, for different detection tasks, the datasets also differ in the types of illness they focus on and language. We show a comprehensive mapping of each method with its associated application using a Sankey diagram (Fig. 3).

#### Data sources

**Fig. 4:**
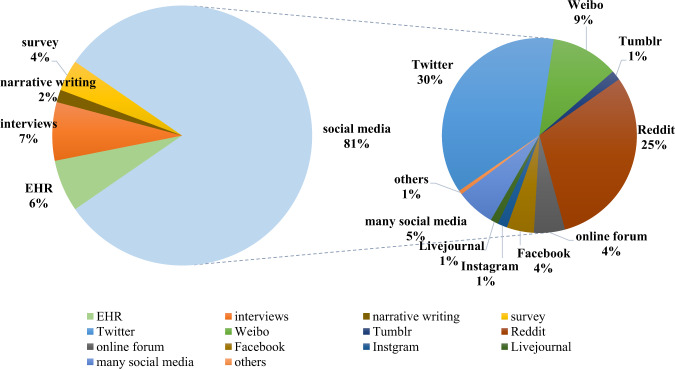
Distribution of different data sources. The pie chart depicts the percentages of different textual data sources based on their numbers.

Figure 4 illustrates the distribution of the different data sources. It can be seen that, among the 399 reviewed papers, social media posts (81%) constitute the majority of sources, followed by interviews (7%), EHRs (6%), screening surveys (4%), and narrative writing (2%).

### Social media posts

The use of social media has become increasingly popular for people to express their emotions and thoughts^20^. In addition, people with mental illness often share their mental states or discuss mental health issues with others through these platforms by posting text messages, photos, videos and other links. Prominent social media platforms are Twitter, Reddit, Tumblr, Chinese microblogs, and other online forums. We briefly introduce some popular social media platforms.

*Twitter.* Twitter is a popular social networking service with over 300 million active users monthly, in which users can post their tweets (the posts on Twitter) or retweet others’ posts. Researchers can collect tweets using available Twitter application programming interfaces (API). For example, Sinha et al. created a manually annotated dataset to identify suicidal ideation in Twitter^21^. Hu et al. used a rule-based approach to label users’ depression status from the Twitter^22^. However, normally Twitter does not allow the texts of downloaded tweets to be publicly shared, only the tweet identifiers—some/many of which may then disappear over time, so many datasets of actual tweets are not made publicly available^23^.

*Reddit*. Reddit is also a popular social media platform for publishing posts and comments. The difference between Reddit and other data sources is that posts are grouped into different subreddits according to the topics (i.e., depression and suicide). Because of Reddit’s open policy, their datasets are publicly available. Yates et al. established a depression dataset named “Reddit Self-reported Depression Diagnosis" (RSDD)^24^, which contains about 9k depressed users and 100k control users. Similarly, CLEF risk 2019 shared task^25^ also proposed an anorexia and self-harm detection task based on the Reddit platform.

*Online forums.* People can discuss their mental health conditions and seek mental help from online forums (also called online communities). There are various forms of online forums, such as chat rooms, discussion rooms (recoveryourlife, endthislife). For example, Saleem et al. designed a psychological distress detection model on 512 discussion threads downloaded from an online forum for veterans^26^. Franz et al. used the text data from TeenHelp.org, an Internet support forum, to train a self-harm detection system^27^.

### Electronic health records

EHRs, a rich source of secondary health care data, have been widely used to document patients’ historical medical records^28^. EHRs often contain several different data types, including patients’ profile information, medications, diagnosis history, images. In addition, most EHRs related to mental illness include clinical notes written in narrative form^29^. Therefore, it is appropriate to use NLP techniques to assist in disease diagnosis on EHRs datasets, such as suicide screening^30^, depressive disorder identification^31^, and mental condition prediction^32^.

### Interviews

Some work has been carried out to detect mental illness by interviewing users and then analyzing the linguistic information extracted from transcribed clinical interviews^33,34^. The main datasets include the DAIC-WoZ depression database^35^ that involves transcriptions of 142 participants, the AViD-Corpus^36^ with 48 participants, and the schizophrenic identification corpus^37^ collected from 109 participants.

### Screening surveys

In order to evaluate participants’ mental health conditions, some researchers post questionnaires for clinician-patient diagnosis of patients or self-measurement. After participants are asked to fill in a survey from crowd-sourcing platforms (like Crowd Flower, Amazon’s Mechanical Turk) or online platforms, the data is collected and labeled. There are different survey contents to measure different psychiatric symptoms. For depression, the PHQ-9 (Patient Health Questionnaire)^38^ or Beck Depression Inventory (BDI) questionnaire^39^ are widely used for assessing the severity of depressive symptoms. The Scale Center for Epidemiological Studies Depression Scale (CES-D) questionnaire^40^ with 20 multiple-choice questions is also designed for testing depression. For suicide ideation, there are many questionnaires such as the Holmes-Rahe Social Readjustment Rating Scale (SRRS)^41^ or the Depressive Symptom Inventory-Suicide Subscale (DSI-SS)^42^.

### Narrative writing

There are other types of texts written for specific experiments, as well as narrative texts that are not published on social media platforms, which we classify as narrative writing. For example, in one study, children were asked to write a story about a time that they had a problem or fought with other people, where researchers then analyzed their personal narrative to detect ASD^43^. In addition, a case study on Greek poetry of the 20th century was carried out for predicting suicidal tendencies^44^.

#### Types of mental illness

**Fig. 5:**
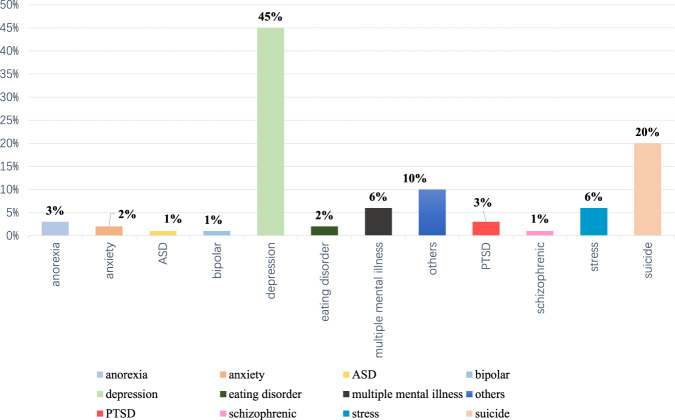
Proportions of various types of mental illness. The chart depicts the percentages of different mental illness types based on their numbers.

There are many applications for the detection of different types of mental illness, where depression (45%) and suicide (20%) account for the largest proportion; stress, anorexia, eating disorders, PTSD, bipolar disorder, anxiety, ASD and schizophrenia have corresponding datasets and have been analyzed using NLP (Fig. 5). This shows that there is a demand for NLP technology in different mental illness detection applications.

The amount of datasets in English dominates (81%), followed by datasets in Chinese (10%), Arabic (1.5%). When using non-English language datasets, the main difference lies in the pre-processing pipline, such as word segmentation, sentence splitting and other language-dependent text processing, while the methods and model architectures are language-agnostic.

### NLP methods used to extract data

#### Machine learning methods

Traditional machine learning methods such as support vector machine (SVM), Adaptive Boosting (AdaBoost), Decision Trees, etc. have been used for NLP downstream tasks. Figure 3 shows that 59% of the methods used for mental illness detection are based on traditional machine learning, typically following a pipeline approach of data pre-processing, feature extraction, modeling, optimization, and evaluation.

In order to train a good ML model, it is important to select the main contributing features, which also help us to find the key predictors of illness. Table 2 shows an overview of commonly used features in machine learning. We further classify these features into linguistic features, statistical features, domain knowledge features, and other auxiliary features. The most frequently used features are mainly based on basic linguistic patterns (Part-of-Speech (POS)^45–47^, Bag-of-words (BoW)^48–50^, Linguistic Inquiry and Word Count (LIWC)^51–53^) and statistics (n-gram^54–56^, term frequency-inverse document frequency (TF-IDF)^57–59^, length of sentences or passages^60–62^) because these features can be easily obtained through text processing tools and are widely used in many NLP tasks. Furthermore, emotion and topic features have been shown empirically to be effective for mental illness detection^63–65^. Domain specific ontologies, dictionaries and social attributes in social networks also have the potential to improve accuracy^65–68^. Research conducted on social media data often leverages other auxiliary features to aid detection, such as social behavioral features^65,69^, user’s profile^70,71^, or time features^72,73^.

**Table 2:** An overview of features used in machine learning-based models.

Feature categories	Feature types	Features	Description	Typical examples
Linguistic features	Sysntactic features	Part-of-Speech (POS)	Based on the grammatical use and functions, words are categorized into different types of POS (like None, Verb, Adverb).	45–47
		Dependency parsing	The grammatical structure of a sentence.	205,206
	Lexicon-based features	Bag-of-words (BoW)	The simplest form of text representation using numbers of vocabularies.	48–50
		Lexical diversity, lexical density	The unique vocabulary usage and proportion of content words.	37
	Emotion features	Sentiment scores	Sentiment scores are used to quantify the feeling of texts and determine the sentiment polarity (positive, negtive,or netural). The way of calculation includes using VADER sentiment analysis (Valence Aware Dictionary and sEntiment Reasoner)^207^, SenticNet 5 lexicon^208^, AFINN lexicon^209^.	63,210–212
		Emotion scores	The emotion scores indicates the user’s emotions and opinions of texts to an extent, which is beneficial for mental issues detection. NRC Affect Intensity Lexicon^213^ are always used.	56, 63, 109,214
	Semantic features	Semantic similarity	Using semantic similarity predict whether the sentence or word is sematically related to the target sentence or word.	60,215
	Topic features	Topic features	The topics extracted from texts using some topic-modeling algorithms, like Latent Dirichlet Allocation (LDA)^216^, Latent Semantic Analysis (LSA)^217^, Non-negative matrix factorization (NMF)^218^.	55, 65, 87,219
	Linguistic features	LIWC	Linguistic Inquiry and Word Count (LIWC)^220^ is always used to automatically extract linguistic styles from texts by calculating the percentages of words in different categories, like linguistic, social affective, etc..	51–53,82
	Others	Hashtag, emoji	Hashtag is metadata tag from social media platform,which present a theme or topic. emoticons or emojis are often used to show various types of emotions.	78, 79,221
Statistical features	Statistical corpus features	n-gram	N-gram is a contiguous sequence of n words.	54–56
		TF-IDF	Term frequency-inverse document frequency (TF-IDF) reflect the importance of the word in document.	57–59,222
		Length statistics	The length of posts, documents or average sentence.	60–62,223
	Vector-based features	Word embedding	The vector-based representation of words. Examples: word2vec^224^, GloVe^118^.	49, 56, 106,225
		Document embedding	The vector-based representation of document.	226
Domain knowledge features	Conceptual features	UMLS	Unified Medical Language System (UMLS) is a set of key terminology, coding standards, and associated resources related to biomedical information.	67,227
		Linguistic dictionary	The dictionary contains mental health illness related words	66, 228,229
Other auxiliary features	Social behavioral features	Social connectivity	The degree of social interaction on social media, like number of followers, friends, and communities joined^230^.	68
		User behaviors	The user’s behavioral signals on social media, such as the frequency of comments and forwardings.	65, 69,231
	Time features	Time features	Focusing on the time-related features, like sending time, time interval.	72,73
	User’s profile features	User’s profile features	The user’s profile features contain their individual information on social networks.	70, 71,231

Machine learning models have been designed based on a combination of various extracted features. The majority of the papers based on machine learning methods used supervised learning, where they described one or more methods employed to detect mental illness: SVM^26,74–77^, Adaptive Boosting (AdaBoost)^71,78–80^, k-Nearest Neighbors (KNN)^38,81–83^, Decision Tree^84–87^, Random Forest^75,88–90^, Logistic Model Tree (LMT)^47,47,91,92^, Naive Bayes (NB)^64,86,93,94^, Logistic Regression^37,95–97^, XGBoost^38,55,98,99^, and some ensemble models combining several methods^75,100–102^. The advantage of such supervised learning lies in the model’s ability to learn patterns from labeled data, thus ensuring better performance. However, labeling the large amount of data at a high quality level is time-consuming and challenging, although there are methods that help reduce the human annotation burden^103^. Thus, we need to use other methods which do not rely on labeled data or need only a small amount of data to train a classifier.

Unsupervised learning methods to discover patterns from unlabeled data, such as clustering data^55,104,105^, or by using LDA topic model^27^. However, in most cases, we can apply these unsupervised models to extract additional features for developing supervised learning classifiers^56,85,106,107^. Across all papers, few papers^108,109^ used semi-supervised learning (models trained from large unlabeled data as additional information), including statistical model ssToT (semi-supervised topic modeling over time)^108^ and classic semi-supervised algorithms (YATSI^110^ and LLGC^111^).

#### Deep learning methods

As mentioned above, machine learning-based models rely heavily on feature engineering and feature extraction. Using deep learning frameworks allows models to capture valuable features automatically without feature engineering, which helps achieve notable improvements^112^. Advances in deep learning methods have brought breakthroughs in many fields including computer vision^113^, NLP^114^, and signal processing^115^. For the task of mental illness detection from text, deep learning techniques have recently attracted more attention and shown better performance compared to machine learning ones^116^.

Deep learning-based frameworks mainly contain two layers: an embedding layer and a classification layer. By using an embedding layer, the inputs are embedded from sparse one-hot encoded vectors (where only one member of a vector is ‘1’, all others are ‘0’, leading to the sparsity) into dense vectors which can preserve semantic and syntactic information such that deep learning models can be better trained^117^. There are many different embedding techniques, such as ELMo, GloVe word embedding^118^, word2vec^119^ and contextual language encoder representations (e.g., bidirectional encoder representations from transformers (BERT)^120^ and ALBERT[121).

According to the structures of different classification layer’s structures, we have divided the deep learning-based methods into the following categories for this review: convolutional neural networks (CNN)-based methods (17%), recurrent neural networks (RNN)-based methods (36%), transformer-based methods (17%) and hybrid-based methods (30%) that combine multiple neural networks with different structures, as shown in Table 3.

**Table 3:** The deep learning methods for mental illness detection.

Type	Method	Description
CNN-based methods	Standard CNN^122–127^	Standard CNN structure: convolutional layer, pooling layer and fully connected layer. Some studies also incorporate other textual features (like POS, LIWC, BoW, etc.).
	Multi-Gated LeakyReLU CNN (MGL-CNN)^128^	Two hierarchical (post-level and user-level)neural network models with gated units and convolutional networks.
	Graph model combined with Convolutional Neural Network^129^	A unified hybrid model combining CNN with factor graph model which leverages social interactions and content.
RNN-based methods	LSTM or GRU (some with attention mechanism)^32,133,136,232–234^	Standard RNN structure: Long Short-Term Memory networks(LSTM) or Gate Recurrent Unit(GRU), and some studies add attention mechanism.
	Hierarchical Attention Network (HAN) with GRU^138^	The GRU with a word-level attention layer and a sentence-level attention layer.
	LSTM with transfer learning^140,141^	Using transfer learning on open dataset for model pre-training.
	LSTM or GRU with multi-task learning^142,235–237^	Using multi-task learning to help illness detection get better result. The tasks include multi-risky behaviors classification, severity score prediction,word vector classification,and sentiment classification.
	LSTM or GRU with reinforcement learning^143,144^	Using reinforcement learning to automatically select the important posts.
	LSTM or GRU with multiple instance learning^145,146^	Using multiple instance learning to get the possibility of post-level labels and improve the prediction of user-level labels.
	SISMO^139^	An ordinal hierarchical LSTM attention model
Transformer-based methods	Self-attention models^148,149^	Using the encoder structure of transformer which has self-attention module.
	BERT-based models (BERT^150,151^, DistilBERT^152^, RoBERTa^153^, ALBERT^150^, BioClinical BERT^31^, XLNET^154^, GPT-1^155^)	Different BERT-based pre-trained models.
Hybrid-based methods	LSTM+CNN^156–160^	Combining LSTM with CNN to extract local features and sequence features.
	STATENet (using transformer and LSTM)^161^	A time-aware transformer combining emotional and historical information.
	Sub-emotion network^164,165,238^	Integrating Bag-of-Sub-Emotion embeddings into LSTM to get emotional information.
	Events and Personality traits for Stress Prediction (EPSP) model^239^	A joint memory network for learning the dynamics of user’s emotions and personality.
	PHASE^166^	A time and phase-aware model that learns historical emotional features from users.
	Hyperbolic graph convolution networks^167^	Hyperbolic Graph Convolutions with the Hawkes process to learn the historical emotional spectrum of a user.

*CNN-based methods.* The standard CNN structure is composed of a convolutional layer and a pooling layer, followed by a fully-connected layer. Some studies^122–127^ utilized standard CNN to construct classification models, and combined other features such as LIWC, TF-IDF, BOW, and POS. In order to capture sentiment information, Rao et al. proposed a hierarchical MGL-CNN model based on CNN^128^. Lin et al. designed a CNN framework combined with a graph model to leverage tweet content and social interaction information^129^.

*RNN-based methods*. The architecture of RNNs allows previous outputs to be used as inputs, which is beneficial when using sequential data such as text. Generally, long short-term memory (LSTM)^130^ and gated recurrent (GRU)^131^ networks models that can deal with the vanishing gradient problem^132^ of the traditional RNN are effectively used in NLP field. There are many studies (e.g.,^133,134^) based on LSTM or GRU, and some of them^135,136^ exploited an attention mechanism^137^ to find significant word information from text. Some also used a hierarchical attention network based on LSTM or GRU structure to better exploit the different-level semantic information^138,139^.

Moreover, many other deep learning strategies are introduced, including transfer learning, multi-task learning, reinforcement learning and multiple instance learning (MIL). Rutowski et al. made use of transfer learning to pre-train a model on an open dataset, and the results illustrated the effectiveness of pre-training^140,141^. Ghosh et al. developed a deep multi-task method^142^ that modeled emotion recognition as a primary task and depression detection as a secondary task. The experimental results showed that multi-task frameworks can improve the performance of all tasks when jointly learning. Reinforcement learning was also used in depression detection^143,144^ to enable the model to pay more attention to useful information rather than noisy data by selecting indicator posts. MIL is a machine learning paradigm, which aims to learn features from bags’ labels of the training set instead of individual labels. Wongkoblap et al. used MIL to predict users with depression task^145,146^.

*Transformer-based methods.* Recently, transformer architectures^147^ were able to solve long-range dependencies using attention and recurrence. Wang et al. proposed the C-Attention network^148^ by using a transformer encoder block with multi-head self-attention and convolution processing. Zhang et al. also presented their TransformerRNN with multi-head self-attention^149^. Additionally, many researchers leveraged transformer-based pre-trained language representation models, including BERT^150,151^, DistilBERT^152^, Roberta^153^, ALBERT^150^, BioClinical BERT for clinical notes^31^, XLNET^154^, and GPT model^155^. The usage and development of these BERT-based models prove the potential value of large-scale pre-training models in the application of mental illness detection.

*Hybrid-based methods.* Some methods combining several neural networks for mental illness detection have been used. For examples, the hybrid frameworks of CNN and LSTM models^156–160^ are able to obtain both local features and long-dependency features, which outperform the individual CNN or LSTM classifiers used individually. Sawhney et al. proposed STATENet^161^, a time-aware model, which contains an individual tweet transformer and a Plutchik-based emotion^162^ transformer to jointly learn the linguistic and emotional patterns. Inspired by the improved performance of using sub-emotions representations^163^, Aragon et al. presented a deep emotion attention model^164^ which consists of sub-emotion embedding, CNN, GRU as well as an attention mechanism, and Lara et al. also proposed Deep Bag of Sub-Emotions (DeepBose) model^165^. Furthermore, Sawhney et al. introduced the PHASE model^166^, which learns the chronological emotional progression of a user by a new time-sensitive emotion LSTM and also Hyperbolic Graph Convolution Networks^167^. It also learns the chronological emotional spectrum of a user by using BERT fine-tuned for emotions as well as a heterogeneous social network graph.

#### Evaluation metrics

Evaluation metrics are used to compare the performance of different models for mental illness detection tasks. Some tasks can be regarded as a classification problem, thus the most widely used standard evaluation metrics are Accuracy (AC), Precision (P), Recall (R), and F1-score (F1)^149,168–170^. Similarly, the area under the ROC curve (AUC-ROC)^60,171,172^ is also used as a classification metric which can measure the true positive rate and false positive rate. In some studies, they can not only detect mental illness, but also score its severity^122,139,155,173^. Therefore, metrics of mean error (e.g., mean absolute error, mean square error, root mean squared error)^173^ and other new metrics (e.g., graded precision, graded recall, average hit rate, average closeness rate, average difference between overall depression levels)^139,174^ are sometimes needed to indicate the difference between the predicted severity and the actual severity in a dataset. Meanwhile, taking into account the timeliness of mental illness detection, where early detection is significant for early prevention, an error metric called early risk detection error was proposed^175^ to measure the delay in decision.

## Discussion

Although promising results have been obtained using both machine and deep learning methods, several challenges remain for the mental illness detection task that require further research. Herein, we introduce some key challenges and future research directions:

- Data volume and quality: Most of the methods covered in this review used supervised learning models. The success of these methods is attributed to the number of training datasets available. These training datasets often require human annotation, which is usually a time-consuming and expensive process. However, in the mental illness detection task, there are not enough annotated public datasets. For training reliable models, the quality of datasets is concerning. Some datasets have annotation bias because the annotators can not confirm a definitive action has taken place in relation to a disorder (e.g., if actual suicide has occurred) and can only label them within the constraints of their predefined annotation rules^9^. In addition, some imbalanced datasets have many negative instances (individuals without mental disorders), which is not conducive to training comprehensive and robust models. Therefore, it is important to explore how to train a detection model by using a small quantity of labeled training data or not using training data. Semi-supervised learning^176^ incorporates few labeled data and large amounts of unlabeled data into the training process, which can be used to facilitate annotation^177^ or improve classification performance when labeled data is scarce. Additionally, unsupervised methods can also be applied in mental disorders detection. For instance, unsupervised topic modeling^178^ increases the explainability of results and aids the extraction of latent features for developing further supervised models.^179,180^
- Performance and instability: There are some causes of model instability, including class imbalance, noisy labels, and extremely long or extremely short text samples text. Performance is not robust when training on the datasets from different data sources due to diverse writing styles and semantic heterogeneity. Thus, the performance of some detection models is not good. With the advances of deep learning techniques, various learning techniques have emerged and accelerated NLP research, such as adversarial training^181^, contrastive learning^182^, joint learning^183^, reinforcement learning^184^ and transfer learning^185^, which can also be utilized for the mental illness detection task. For example, pre-trained Transformer-based models can be transferred to anorexia detection in Spanish^186^, and reinforcement networks can be used to find the sentence that best reflects the mental state. Other emerging techniques like attention mechanism^187^, knowledge graph^188^, and commonsense reasoning^189^, can also be introduced for textual feature extraction. In addition, feature enrichment and data augmentation^190^ are useful to achieve comparable results. For example, many studies use multi-modal data resources, such as image^191–193^, and audio^194–196^, which perform better than the single-modal text-based model.
- Interpretability: The goal of representation learning for mental health is to understand the causes or explanatory factors of mental illness in order to boost detection performance and empower decision-making. The evaluation of a successful model does not only rely on performance, but also on its interpretability^197^, which is significant for guiding clinicians to understand not only what has been extracted from text but the reasoning underlying some prediction^198–200^. Deep learning-based methods achieve good performance by utilizing feature extraction and complex neural network structures for illness detection. Nevertheless, they are still treated as black boxes^201^ and fail to explain the predictions. Therefore, in future work, the explainability of the deep learning models will become an important research direction.
- Ethical considerations: It is of greater importance to discuss ethical concerns when using mental health-related textual data, since the privacy and security of personal data is significant and health data is particularly sensitive. During the research, the researchers should follow strict protocols similar to the guidelines^202^ introduced by Bentan et al., to ensure the data is properly applied in healthcare research while protecting privacy to avoid further psychological distress. Furthermore, when using some publicly available data, researchers need to acquire ethical approvals from institutional review boards and human research ethics committees^203,204^.

There has been growing research interest in the detection of mental illness from text. Early detection of mental disorders is an important and effective way to improve mental health diagnosis. In our review, we report the latest research trends, cover different data sources and illness types, and summarize existing machine learning methods and deep learning methods used on this task.

We find that there are many applications for different data sources, mental illnesses, even languages, which shows the importance and value of the task. Our findings also indicate that deep learning methods now receive more attention and perform better than traditional machine learning methods.

We discuss some challenges and propose some future directions. In the future, the development of new methods including different learning strategies, novel deep learning paradigms, interpretable models and multi-modal methods will support mental illness detection, with an emphasis on interpretability being crucial for uptake of detection applications by clinicians.

### Reporting summary

Further information on research design is available in the Nature Research Reporting Summary linked to this article.

## Supplementary information

Reporting Summary

## Data Availability

The data that support the findings of this study are available from the corresponding author upon reasonable request.
